# P-SILI as justification for intubation in COVID-19: readers as arbiters

**DOI:** 10.1186/s13613-020-00774-5

**Published:** 2020-11-18

**Authors:** Martin J. Tobin, Amal Jubran, Franco Laghi

**Affiliations:** Division of Pulmonary and Critical Care Medicine, Hines Veterans Affairs Hospital and Loyola University of Chicago Stritch School of Medicine, Hines, IL 60141 USA

## Dear Editor,

We thank Dr. Gattinoni and colleagues for their response [1] to our letter [2].

Before addressing scientific matters now raised, we wish to acknowledge the enormous research contributions of Dr. Gattinoni and colleagues. It is because Dr. Gattinoni is so highly regarded that his opinions concerning the role of patient self-inflicted lung injury (P-SILI) as warrant for initiating mechanical ventilation (during a pandemic with no end in sight) demand careful scrutiny.

The ultimate arbiter in this exchange is the reader. Readers need to see countervailing arguments laid out and then draw their own conclusions. Scientific debates are often arcane or arid, but the endpoint in the present dialectic engagement is deadly important: the most effective treatment for patients rendered extremely vulnerable by an ongoing pandemic. P-SILI has been put forward as justification for early intubation in preference to noninvasive strategies not only by Gattinoni and colleagues, but also by the highly influential WHO Guidelines [3].

Dr. Gattinoni and colleagues refer to an article by Stalcup and Mellins on pulmonary edema consequent to inspiratory efforts in patients with asthma. It was common knowledge among physiology researchers in the mid 1980s that the data in this report were not to be trusted. Stalcup and his co-investigators retracted at least seven articles published in *Journal of Applied Physiology, Journal of Clinical Investigation* and elsewhere. Journals retract articles only at the request of authors; when several papers have been retracted, it is imprudent to rely on data in other studies by that author, which Dr. Gattinoni and colleagues are recommending readers to treat as valid information.

Gattinoni and colleagues focus on a sentence from an editorial by Dr. Tobin: “The surest way to increase COVID-19 mortality is liberal use of intubation and mechanical ventilation” [4]. We stand by this sentence and believe data with the pandemic supports the wisdom of the advice [5, 6]. Emerging data from the Intensive Care National Audit & Research Centre (ICNAARC) reveal that 28-day mortality of COVID-19 patients admitted to the ICU decreased from 43.5% (95% CI 41.6% to 45.5%) for the time period 1 February–28 March to 34.4% (95% CI 32.3% to 36.2%) for time period 16 April–21 May, 2020 [6]. Over the same period, the rate of intubation (and mechanical ventilation) decreased from 75.9 to 44.1% [6].

Dr. Gattinoni and colleagues have presented their beliefs on the importance of P-SILI, we have presented our counterarguments. It is for each reader to answer “Is there sufficient scientific evidence to say that P-SILI exists in patients and that it serves as justification for endotracheal intubation of Covid patients?”.

## Data Availability

Not applicable.

## Abbreviations

CI: Confidence interval
COVID-19: Coronavirus Disease 2019
ICNAARC: Intensive Care National Audit & Research Centre
P-SILI: Patient self-induced lung injury
WHO: World Health Organization
